# Engineering of TeO_2_-ZnO-BaO-Based Glasses for Mid-Infrared Transmitting Optics

**DOI:** 10.3390/ma13245829

**Published:** 2020-12-21

**Authors:** Kadathala Linganna, Jung-Hwan In, Seon Hoon Kim, Karam Han, Ju Hyeon Choi

**Affiliations:** Intelligent Optical Module Research Center, Korea Photonics Technology Institute, Gwangju 61007, Korea; injh15@kopti.re.kr (J.-H.I.); shkim@kopti.re.kr (S.H.K.); rkfka8811@kopti.re.kr (K.H.)

**Keywords:** mid-IR optical glass, thermal stability, coefficient of thermal expansion, hardness, refractive, transmittance

## Abstract

In this paper, the glass systems, TeO_2_–ZnO–BaO (TZB), TeO_2_–ZnO–BaO–Nb_2_O_5_ (TZB–Nb) and TeO_2_–ZnO–BaO–MoO_3_ (TZB–Mo), were fabricated by the traditional melt-quench protocol for use as mid-infrared (mid-IR) transmitting optical material. The effect of Nb_2_O_5_ and MoO_3_ on the key glass material properties was studied through various techniques. From the Raman analysis, it was found that the structural modification was clear with the addition of both Nb_2_O_5_ and MoO_3_ in the TZB system. The transmittance of studied glasses was measured and found that the optical window covered a region from 0.4 to 6 μm. The larger linear refractive index was obtained for the Nb_2_O_5_-doped TZB glass system than that of other studied systems. High glass transition temperature, low thermo-mechanical coefficient and high Knoop hardness were noticed in the Nb_2_O_5_-doped TZB glass system due to the increase in cross-linking density and rigidity in the tellurite network. The results suggest that the Nb_2_O_5_-doped TZB optical glasses could be a promising material for mid-infrared transmitting optics.

## 1. Introduction

In recent years, interest on infrared optical systems has been increased because of their extensive usage in optical fields. These optical systems usually use the electromagnetic radiations in atmospheric window of mid-infrared region covering from 3 to 12 μm [1,2,3,4]. As per reported literature, the infrared crystalline materials have been conventionally deployed to fabricate infrared lenses [5,6,7]. However, the evolution of optical systems using crystalline materials possesses drawbacks like being time-consuming, expensive and very difficult for mass production.

In this regard, the amorphous chalcogenide glass systems have been investigated rigorously for the development of infrared optical systems [8,9]. However, these glasses have drawbacks like low level of transmission, poor thermal stability and prone to crystallization, leading to investigate the alternative mid-infrared transmitting optical glasses. Among soft glass systems, tellurium oxide (TeO_2_)-based glasses are emerging as enabling materials for mid-infrared (IR) optics due to their wide array of functional properties, such as wide transmission ranging from ultraviolet (UV) to mid-IR (0.4 to 6 μm), low melting temperatures (~800 °C), good thermal stability (≥100 °C), larger index of refraction (≥2.0), low maximum phonon energies (~750 cm^−1^) and larger Raman gain coefficient [10,11,12,13]. Many researchers have thus studied the tellurite glass materials as contenders for a range of optical applications that include lasers/amplifiers [14,15,16,17,18,19,20], ceramic bulk lasers [21], upconverters [22,23], Raman amplifiers [24,25], mid-infrared lasers [26] and non-linear optical devices [27].

From the investigations, it was noted that key glass material properties that include thermal, thermo-mechanical, mechanical and optical are important and they must be known for precision glass molding/fiber processes of optical glasses. In particular, for the application of mid-infrared transmitting optics, the most important parameters are the transformation temperature (T_g_), the softening temperature (T_s_) and the thermo-mechanical coefficient (α) that play an important role, therefore, they must be studied in dependence of glass composition. In particular, the data of T_s_ and α were not available for many studied glass compositions. The tuned glass-forming area is required in order to establish a relationship between physical and optical properties and glass composition.

Hence, the present study is targeted to develop a high index of refraction (>1.8 at 3, 4 and 5 μm), high transmittance (>70%), low glass transition temperature (≤450 °C), low coefficient of thermal expansion (≤15 × 10^−6^/K at 100 °C) and good mechanical stability (>300 kgf/mm^2^) glass material for the design of a lens. Many researchers have already reported the TeO_2_–ZnO–based glass systems for various optical applications [10,12,15,17,19,24,27]. However, the TeO_2_–ZnO glass system showed low thermal and optical properties [10,12,15,17,19,24,27]. Thus, we have chosen the TeO_2_–ZnO–BaO glass system for the design and evolution of mid-infrared glass lens through the glass molding process. The effect of Nb_2_O_5_ and MoO_3_ additions on key glass properties in the TeO_2_–ZnO–BaO glass system was investigated. The addition of Nb_2_O_5_ and MoO_3_ transition metal oxides into the network of tellurite strengthens the connectivity and improves the glass forming ability (GFA) as well, which leads to improve the physical and optical properties [28].

## 2. Materials and Methods

**Figure 1:**
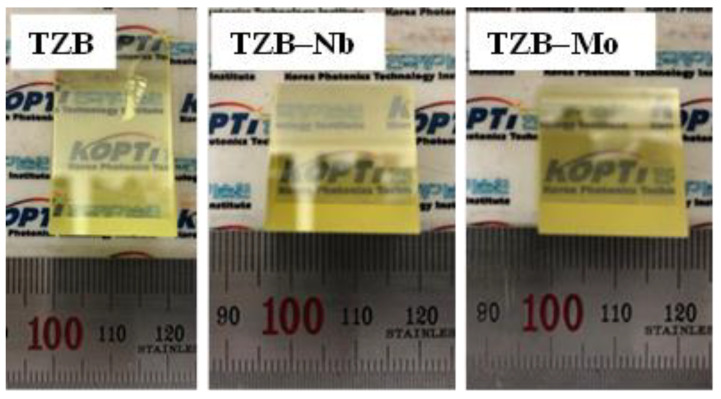
Photographs of indigenously developed TeO_2_-ZnO-BaO TZB series prism samples.

Reagent grade TeO_2_, ZnO, BaO, Nb_2_O_5_ and MoO_3_ chemical constituents with purity of 99.99% were used for the preparation of glasses, and the molar ratio of glasses is shown in Table 1. The net weight of 30 g of raw materials was thoroughly grinded using a ball mill (PL-BM5L, Poong Lim, Seoul, South Korea) for 1 h and then mixed powder was taken in Pt crucible and heated at 900 °C using an electric furnace in a flowing N_2_ atmosphere for 45 min. The melt was poured onto a brass plate that was preheated and then the glass samples were annealed near the T_g_ for 2 h and naturally cooled down to room temperature. The glass samples free of defects were optically polished glasses and were inspected for the measurement. Figure 1 displays the photographs of indigenously developed TZB series prism samples.

The Raman experiment for the fabricated tellurite glass samples was conducted by using the Horiba Jobin-Yvon, France LabRam HR800 UV Raman spectrometer. The Raman spectra were recorded in the frequency region of 200–1200 cm^−1^ by focusing an Ar ion laser at 514.5 nm. The laser exposure time of 10 s, accumulation time of 3× and objective lens (100×, numerical aperture (N.A) = 0.85), were applied for obtaining the data. At room temperature (RT), the vibrational spectra were recorded with a resolution of 0.5 cm^−1^ in an unpolarized mode and backscattering geometry. The optical transmission spectra were measured by Frontier Optica Fourier-Transform Infrared (FTIR) Spectrometer (Perkin Elmer, L1280032, Cleveland, OH, USA) in the wavelength region from 0.4 to 6.0 μm. The glass samples with thickness of 2 mm were used for the measurement. Refractive index of TZB and TZB–10Nb glasses was measured by SpectroMaster@HR (Trioptics, Wedel, Germany) at different mid-infrared wavelengths of 3, 4 and 5 μm. In order to get high accuracy in the measurement, the glass block of 40 × 40 × 40 mm^3^ was cleaved into a prism shape and then polished. The accuracy of the instrument for single measurement was found to be 0.2 arcsec. In this study, a HgCdTe detector was used for the mid-IR wavelengths of 3, 4 and 5 μm. The simultaneous thermal analyzer (STA 409, NETZSCH, Wittelsbacherstraße, Selb, Germany) was deployed for the calorimetric analysis. For the measurement, about 25 mg glass powder was taken in the alumina pan and then the thermal traces were measured in the range from RT to 800 °C at a rate of 10 K/min under nitrogen atmosphere. The dilatometer (DIL 402C, NETZSCH, Wittelsbacherstraße, Selb, Germany) was used for measuring the dilatometric traces of glass samples with size of 5 × 5 × 10 mm^3^ in the range of 30 to 450 °C. The fitting accuracy of thermal expansion was 0.1 K. Micro-hardness of the resultant glasses were determined by a hardness tester (Mitutoyo HM-200, Aurora, IL, USA). The glass samples with size of 5 × 5 × 10 mm^3^ were used for measuring the hardness.

## 3. Results and Discussion

### 3.1. Structural Analysis—Raman Spectra

**Figure 2:**
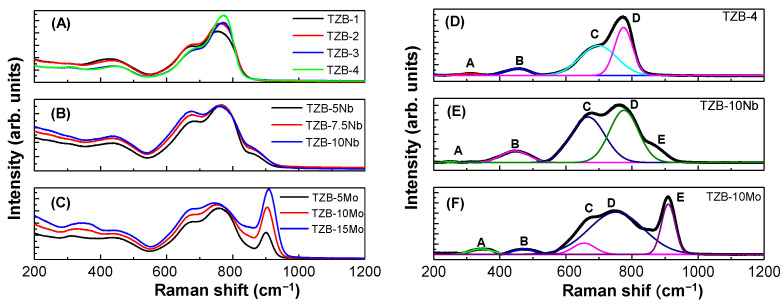
(**A**–**C**) Raman spectra of TZB, TZB−Nb and TZB−Mo glass systems and (**D**–**F**) Deconvoluted Raman spectra of TZB−4, TZB−10Nb and TZB−10Mo glasses using Gaussian fit.

The characterization of structural behavior for the titled glasses using vibrational spectroscopy is vital for optical device application. Figure 2A–C displays the Raman spectra of the TZB glass system for different modifier concentrations. Figure 2D–F depicts the deconvoluted Raman spectra of one of the characteristic glass compositions of each series compositions. It was found that the observed Raman bands of the presented glasses are corresponding to the same structural units of α–TeO_2_ crystal and tellurite glasses containing various modifier oxides [10,11,13,29,30,31,32,33]. As described in the literature [10,11,29,30,31,32,33], the tellurite network possesses a basic structural fragment of TeO_4_ trigonal bipyramid (tbp), TeO_3_ trigonal pyramid (tp) and intermediate TeO_3+δ_ polyhedron. In the present study, the vibrational bands were noted in the frequency region of 309–304, 428–452, 671–692 and 754–771 cm^−1^ for the TZB glass system at different BaO contents, as shown in Figure 2A. The Raman bands’ frequency and its assignment are shown in Table 2. The deconvoluted Raman spectrum for the characteristic glass composition in this series is shown in Figure 2D. As is seen, the amplitude of 300, 428 and 671 cm^−1^ frequency bands decreased, while the 754 cm^−1^ frequency band increased with the increase in BaO content substituted for TeO_2_, indicating that the number of bridging oxygen’s decreases, which in turn reduces the network connectivity. The enhancement of the band at ~754 cm^−1^ was due to the increase of ν(Te–O) stretching and bending vibrations in TeO_3+δ_ and TeO_3_ networks that occur with the addition of BaO. It was also noted that the width of the Raman bands decreased with the addition of BaO content. This clearly determines the change of TeO_4_ tbp structural fragment into TeO_3+δ_ or TeO_3_ units with the addition of BaO content.

The Raman spectra for the TZB−Nb glasses at different Nb_2_O_5_ concentrations are depicted in Figure 2B. As could be seen from the spectra, five characteristic vibrational frequency bands were found in the range of 308–310, 439–440, 681–676, 761–758 and 865–851 cm^−1^. The optical phonon frequencies and assignment are tabulated in Table 2 and the identified bands were similar to the Nb_2_O_5_-based tellurite glasses reported elsewhere [34,35,36,37,38]. The deconvoluted Raman spectrum for the representative glass in this series is shown in Figure 2E. The comparison of the Raman spectra of Nb_2_O_5_-doped TZB glasses showed that the intensity of Te–O stretching and bending vibrations in TeO_3+δ_ and TeO_3_ networks at 310 cm^−1^ decreased with respect to the Nb_2_O_5_ concentration. The frequency bands at 439, 681 and 761 cm^−1^ attributed to the ν_s_(Te−O−Te) stretching and bending, ν_as_(Te–O) stretching, and ν(Te−O) stretching vibrations increased with the addition of Nb_2_O_5_ content, respectively. The band centered at ~865 cm^−1^ was noticed in the higher frequency region, which can be assigned to the ν_s_(Nb–O) stretching vibrations in NbO_6_ octahedra [34,35,36,37,38], and its intensity decreased with the addition of Nb_2_O_5_ concentration. The position of all the major bands moved towards lower wave numbers from 681 to 676 cm^−1^, 761 to 758 cm^−1^ and 865 to 851 cm^−1^ respectively, with the increase in Nb_2_O_5_ additive concentration. Thus, the structural change occurs from TeO_4_ tbp units into TeO_3_/TeO_3+δ_ units along with the creation of non-brdging oxygens (NBOs), as the Nb_2_O_5_ content increases. This structural rearrangement determines that the tellurite network adopted the maximum number of Nb^5+^ ions as modifier ions [39]. It was worthy to note that the Raman spectral bandwidth of all the bands of Nb_2_O_5_-based TZB glasses was found to be larger that of the bands identified for base TZB glass compositions. In view of the bandwidth, the TZB−Nb glasses could be useful for the broadband Raman amplifier application.

The Raman spectra for the TZB–Mo glasses at different MoO_3_ concentrations are depicted in Figure 2C. As could be seen from the spectra, five characteristic frequency bands were noticed in the range of 304–338, 433–436, 678–679, 754–745 and 900–909 cm^−1^. The assignment and peak positions of frequency bands are tabulated in Table 2 and the identified bands are similar to the MoO_3_-based tellurite glasses reported elsewhere [40,41,42,43]. Overall, the spectra exhibited two continuous broad bands, which are composed of the characteristic vibration bands of Te−O polyhedron, Mo–O polyhedron and possible Te−O–Mo bonds. The deconvoluted Raman spectrum for the representative glass composition in this series is depicted in Figure 2F. The accuracy of fitting was found to be 99.9%. Five characteristic frequency bands of Te−O and Mo−O groups were identified at 344, 468, 655, 752 and 910 cm^−1^ and are similar to the reported TeO_2_−MoO_3_ glass systems [40,41,42,43]. As is seen from Figure 2C, the intensity of all frequency bands increased with the MoO_3_ additive content. In addition, the shift in peak position of bands with respect to MoO_3_ concentration was noticed. Peak A from 304 to 338 cm^−1^, peak B from 433 to 436 cm^−1^, peak C from 678 to 679 cm^−1^, peak D from 754 to 745 cm^−1^ and peak E from 900 to 909 cm^−1^ shifted with an increase in MoO_3_ content. This shows a change in bond length of the frequency group and a distortion of the basic TeO_4_ tbp structural unit, resulting in a variation of physical and optical properties.

### 3.2. Optical Studies

#### 3.2.1. Mid-Infrared Optical Transmittance

**Figure 3:**
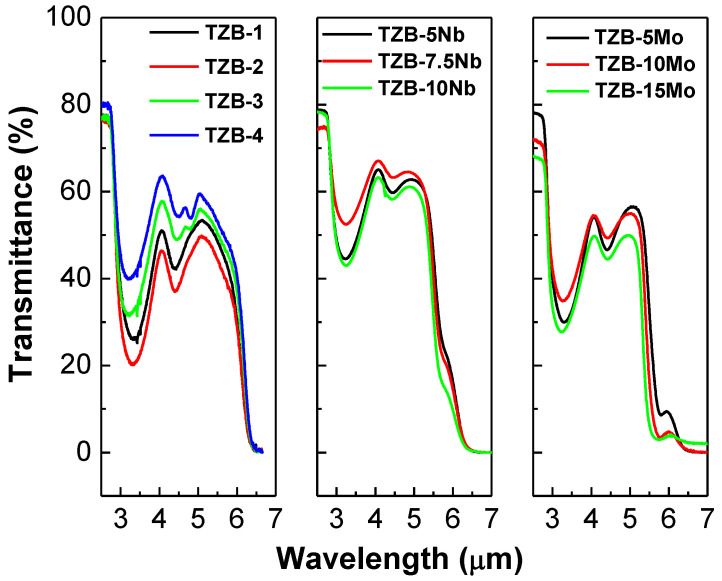
Mid-infrared (IR) transmission spectra of TeO_2_−BaO−ZnO glasses varied with different additive concentrations.

Figure 3 shows the transmittance of all three series glasses in the mid-IR region ranging from 3 to 6 μm. It was clear that the titled glasses added with different additives showed a good transmittance up to 6 μm, indicating attractive mid-infrared transmitting optics. As is seen from transmission spectra, the transmission level of all the samples was close to 70%. The broad absorption bands were due to the absorption of glass matrix, Fresnel reflections and dispersion [44,45,46,47,48]. At ~3 μm, an intense hollow was observed, which corresponds to free hydroxyl groups, and the weaker hollow at ~4.5 μm attributes to multiphonon absorption [44,45,46,47,48]. The hydroxyl group absorption can be removed in the tellurite glasses with addition of fluorides along with sealing gas (Ar or O_2_) [44,45,46,47,48].

The coefficient of absorption (α_OH−_) and OH−concentration (N_OH−_) in the resultant tellurite glasses can be obtained by the following two equations [44,45,46,47,48], given by:

(1) $$\alpha_{\mathrm{OH}-}=\frac{\ln\left(\frac{T_{0}}{T}\right)}{L}$$

(2) $$N_{\mathrm{OH}-}=\frac{N_{\mathrm{AV}}}{\varepsilon }\alpha_{\mathrm{OH}-}$$

where L is the thickness (cm), T_0_ is the maximum transmittance, T is the transmittance at ~3 μm, N_AV_ is the Avogadro constant (6.02 × 10^23^ mol^−1^) and ε is the molar absorptivity corresponding to OH^−^ in tellurite glasses (49.1 × 10^3^ cm^2^/mol) [49]. The α_OH−_ for TZB series glasses was calculated to be 5.44, 6.56, 4.43 and 3.51 cm^−1^, respectively. The α_OH−_ of Nb_2_O_5_-doped TZB glasses was found to be 2.85, 1.76 and 3.03 cm^−1^, respectively. The α_OH−_ of MoO_3_-doped TZB glasses was found to be 4.47, 3.62 and 4.49 cm^−1^, respectively. The N_OH−_ values were obtained to be 6.67, 8.04, 5.43 and 4.30 (×10^19^ cm^−3^) for different BaO contents of 5, 10, 15 and 20 mol%, respectively. The N_OH−_ values were calculated to be 3.49, 2.16 and 3.71 (×10^19^ cm^−3^) for different Nb_2_O_5_ contents of 5, 7.5 and 10 mol%, respectively. The N_OH−_ values were found to be 3.49, 2.16 and 3.71 (×10^19^ cm^−3^) for different MoO_3_ contents of 5, 10 and 15 mol%, respectively. Among all three series glasses, it was clear that the Nb_2_O_5_-doped TZB glasses showed lower values of α_OH−_ and N_OH−_. The α_OH−_ and N_OH−_ values are comparable to the reported oxyfluoride tellurite glasses [47]. From the results, it was suggested that the Nb_2_O_5_-doped TZB glasses could be incorporated with fluorides in order to reduce more OH− absorption in the glass network.

#### 3.2.2. Refractive Index and Optical Dispersion

**Figure 4:**
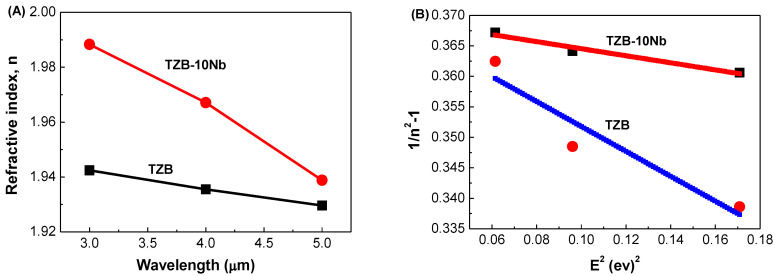
(**A**) Variation of refractive index in dependence of wavelength for TZB and TZB–10Nb glasses. (**B**) Variation of refractive index with photon energy, obtained using Wemple’s approximation for the experimental data of TZB and TZB–10Nb glasses. The red and blue curves indicate the two terms Sellmeier equation fit to the data.

In addition to transmittance, the index of refraction and dispersion are vital parameters for the application of optical materials in optical device systems. The refractive index was measured in the mid-IR region at 3.0, 4.0 and 5.0 μm for the TZB and TZB–Nb glasses (see Figure 4A). From the figure, as usual, the refractive index decreases with respect to wavelength, irrespective of the glass composition. With the addition of Nb_2_O_5_ to the base TZB glass system, the refractive index increased significantly, as shown in Figure 4A. The reason for this behavior can be explained based on the oxide ion polarizability. Dimitrov et al. [50] have reported that the cation polarizability of oxides (α_O2−_) affects the refractive index in the order of Te^4+^ (1.595 Å^3^) > Nb^5+^ (1.035 Å^3^) > Zn^2+^ (0.286 Å^3^). In the present study, the ZnO content was replaced with Nb_2_O_5_, resulting in a high refractive index for the Nb^5+^-doped glasses. Based on the oxide ion polarizability of simple oxides, an optical basicity (Λ) was obtained from the relation, Λ = 1.67(1−1/α_O2−_). The optical basicity (Λ) of TeO_2_, Nb_2_O_5_ and ZnO was reported to be 0.93, 1.05 and 1.08, respectively [50]. The effect of Nb_2_O_5_ addition on Abbe number was studied for the TZB glass system. The Abbe number is used in order to know dispersion of optical materials. Abbe number (ν) can be calculated from the following equation [51], given by:

(3) $$\nu \;=\;\frac{n_{4}-1}{n_{3}-n_{5}}$$

where n_3_, n_4_ and n_5_ represent the refractive indices at wavelengths 3, 4 and 5 μm, respectively. The ν for the TZB and Nb-doped TZB glasses was found to be 73.08 and 19.53, respectively. This indicates that the optical dispersion depends on the glass composition. From the results, the Nb-doped TZB glasses are considered as high dispersive material. The variation of refractive index with photon energy can be obtained based on the single oscillator approximation suggested by Wemple [52]:

(4) $$n^{2}-1=\;\frac{E_{d}E_{o}}{E_{o}^{2}-E^{2}}\;\frac{1}{n^{2}-1}=\frac{E_{o}}{E_{d}}-\frac{E^{2}}{E_{o}E_{d}}$$

where n is the refractive index at a specific wavelength, E is the photon energy (= hν), E_o_ is the average excitation energy for electronic transitions and E_d_ is the dispersion energy. E_o_ and E_d_ values for the TZB and TZB–Nb glasses were determined from the linear fit of 1/(n^2^ − 1) vs E^2^ plot, as shown in Figure 4B. The E_o_ and E_d_ values were found to be 2.5285 and 6.8278 for TZB glasses, while 1.3491 and 3.6243 values were obtained for the TZB–10Nb glass, respectively. In single crystal, the dispersion energy, E_d_, depends on the neighbor cation coordination number and anion valency. In the present study, the addition of Nb_2_O_5_ contents shows a decrease in the E_d_ values. In case of glasses, the E_d_ value follows a similar trend but lower than that of the values for single crystal [52]. According to the reported literature, the 5% addition of Nb_2_O_5_ to the Na_2_O–TeO_2_ system results in an increase of the E_d_ value up to 70% [53]. This may probably be due to the substitution of large ionic radii atoms like Na^+^, that are also likely to influence the optical characteristics [54]. A decrease in dispersion energies, E_d_, with the composition infers the decrease in the covalency of glass network with the increase of Nb content.

### 3.3. Thermal Studies

**Figure 5:**
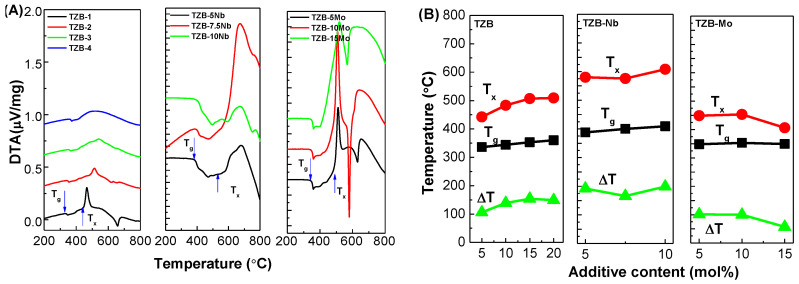
Differential Scanning Calorimetric (DSC) curves (**A**) and Quantitative data (**B**) of the TeO_2_–ZnO–BaO glass system varied with different additive concentrations.

The calorimetric study is used to investigate the thermal stability of glass. The thermal stability, i.e., ΔT = T_x_ − T_g_, should be as high as possible to have a wide working range. The larger the thermal stability, the greater the glass quality. The typical ΔT should be ≥100 °C to avoid crystal formation inside the glass. The fiber drawing and glass molding processes require high ΔT as the optical glasses undergo repeated reheating cycles. Figure 5A represents the qualitative calorimetric data of the TeO_2_–ZnO–BaO glass system incorporated with different additives of BaO, Nb_2_O_5_ and MoO_3_. From the calorimetric analysis, the characteristic temperatures such as T_g_, T_x_ and ∆T were obtained for the resultant glasses. It was noted that all the glasses exhibited single glass transition. The inferred values of these temperatures are tabulated in Table 3 and depicted in Figure 5B. The T_g_ and T_x_ increased from 336 to 360 °C and 442 to 509 °C with the addition of BaO content, respectively. The trend of T_g_ could be attributed to the increased cross-linking density and bond strength between the atoms involved [10]. This can be explained based on the ionic radii of Te (0.89 Å) and Ba (1.45 Å). The substitution of BaO, instead of TeO_2_, leads to cleaving Te–O–Te linkages and filling open spaces due to its high ionic radii compared to that of Te^4+^ [10]. It was worthy to note that the intensity of the T_x_ decreased with the increase in BaO content and it almost vanished at 20 mol% of BaO owing to structural rearrangement. As can be seen from Figure 5B, ΔT increased with the substitution of BaO instead of TeO_2_ (Table 3).

In case of TZB–Nb, with the addition of Nb_2_O_5_, the parameters T_g_ and T_x_ showed an increasing trend from 388 to 409 °C and 580 to 598 °C, respectively. There are two reasons for this behavior in Nb_2_O_5_-modified glasses. In the niobium-tellurite glass network, Nb^5+^ ions exist partially in both NbO_6_ octahedra (mainly connect to non-bridging oxygen, NBO), and NbO_4_ tetrahedra (mainly connect to bridging oxygen, BO) [33,37,39]. Moreover, the bond energy of Nb^5+^ (771 kJ/mol) was larger than that of Zn^2+^ (151 kJ/mol) [37,39,50]. This shows that the bond cleaving in the Nb_2_O_5_–TeO_2_ glass network becomes difficult, leading to an increase in T_g_ and T_x_. It was interesting to note from Figure 5A that the crystallization peaks were weak for the TZB–10Nb glass, indicating that the glass sample was more stable against devitrification and thus appropriate for the glass molding process/fiber fabrication.

For the TZB–Mo glass system, the T_g_ and T_x_ values were determined to be in the range of 350 to 352 °C and 487 to 408 °C, respectively. The considerable variation was not noticed for the T_g_ with the addition of MoO_3_, while the T_x_ was found to decrease with the MoO_3_ addition. The trend of the thermal characteristics of TZB–Mo glasses can be explained based on structural behavior. The substitution of MoO_3_ instead of ZnO into the tellurite network breaks the Te–O bonds, decreases the number of bridging oxygens and encourages the creation of Te−O−Mo bonds in the present glass. Additionally, the transformation of TeO_4_ tbp units into TeO_3+δ_/TeO_3_ units eventually leads to a more disordered structure, resulting in the deteriorated or even vanished peak of crystallization. This conclusion was sustained by Raman studies which confirmed that the frequency band at 900 cm^−1^ had dominant behavior compared to that of 750 cm^−1^.

It was interesting to note that the studied TZB-based glass systems performed the key glass properties (T_g_, T_s_, α and H_K_) that were comparable to the commercial SUMITA optical glasses. The commercial SUMITA Optical Glass MFG. Co., Ltd. Company (Saitama, Japan) had developed the mid-IR optical glasses (K-FIR98UV, K-FIR98UV) with specific values of thermal properties for precision glass molding. The quantitative values of T_g_, T_s_, α and H_K_ were reported to be 427, 447 °C, 16.3 × 10^−6^/K and 351 kgf/mm^2^, respectively. In particular, the Nb_2_O_5_-based TZB glasses showed close values to the commercial glasses and thus they are promising candidates for glass molding.

### 3.4. Thermo-Mechanical Studies

**Figure 6:**
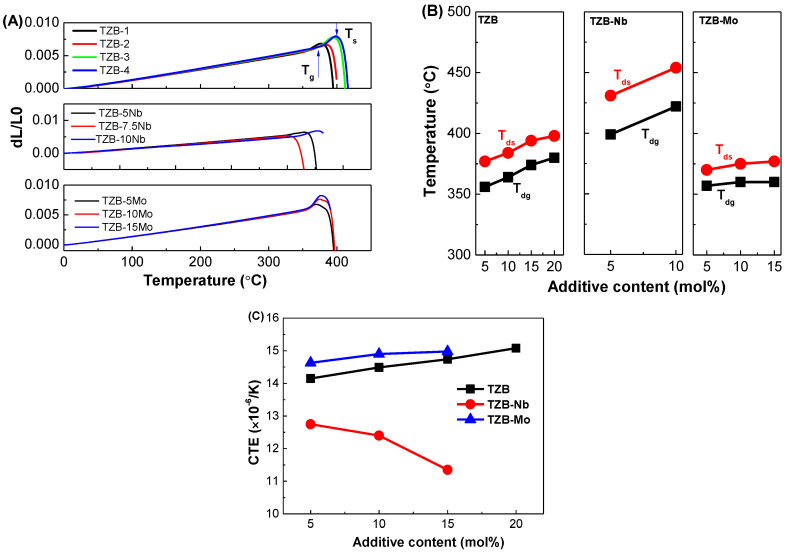
(**A**) Dilatometric curves, (**B**) Quantitative data of dilatometric glass transition temperature (T_dg_) and dilatometric softening temperature (T_ds_), and (**C**) Coefficient of thermal expansion (CTE) of the TeO_2_–ZnO–BaO glass system varied with different additive concentrations.

The thermo-optic coefficient (CTE, α) is a vital parameter for the realization of several practical optical systems [55]. The α can lead to stress-optic effects and also influences the thermo-optic coefficient, dn/dT of glass [56,57,58]. The dilatometer was used for measuring the dilatometric traces of titled glasses in order to obtain the properties like dilatometric glass transition temperature (T_dg_), dilatometric softening temperature (T_ds_) and α. Figure 6A displays the qualitative dilatometric traces of studied glasses. The α was determined by linear fitting of the curves measured in the temperature range of 25 to 450 °C. The T_dg_ was determined from the expansion curve using the interception method, while the T_ds_ was determined by the maximum temperature of the expansion curve. The quantitative values of T_dg_ and T_ds_ for the resultant glasses are shown in Figure 6B and Table 3. The quantitative values of α for the resultant glasses are shown in Figure 6C and Table 3. For the TZB series glasses, the α increased from 14.15 to 15.05 (×10^−6^/K at 100 °C) with respect to BaO content varied from 5 to 20 mol%, respectively. The T_dg_ and T_ds_ temperatures also increased from 356 to 380 °C and 377 to 398 °C with respect to BaO content, respectively. To understand the trend of α for the studied glasses, as shown in Figure 6C, we consider the electronegativities of the modifying cations. The relative electronegativities of elements determine the strength of the chemical bonds between them. As such, when discussing a substitution of one element for another in a glass matrix, the electronegativities of the elements being exchanged are directly related to the bond strengths. The trend of the α can also be explained based on the field strength of cations and the molecular weight of modifiers. For the TZB system, the α increased with the increase in BaO content instead of TeO_2_ due to an increase of the molecular weight of BaO.

For the TZB–Nb system, the α decreased from 12.75 to 11.35 (×10^−6^/K at 100 °C) with the increase in doping of Nb_2_O_5_, respectively. The T_dg_ and T_ds_ temperatures increased from 399 to 422 °C and 431 to 454 °C respectively, maybe due to higher single bond strength of the Nb–O bond (~771 kJ/mol) than that of Zn–O (~151 kJ/mol) and Te–O bonds (~285 kJ/mol) [50]. The increased network strength realized through Nb_2_O_5_ addition holds the glass together more strongly and thus lowers the glass response to thermal variations. Based on the relative electronegativities of Nb^5+^ (1.6) and Zn^2+^ (1.65), the bond strength increases when Nb^5+^ is substituted for Zn^2+^ in the glass network. Accordingly, the α decreased for the TZB–Nb glass system for different Nb_2_O_5_ contents. The α (14.63 to 14.98 × 10^−6^/K at 100 °C), T_dg_ (357 to 360 °C) and T_dg_ (370 to 377 °C) increased with the substitution of MoO_3_ content, respectively. Based on the relative electronegativities of Mo^6+^ (2.15) and Zn^2+^ (1.65), the bond strength decreases when Mo^6+^ is substituted for Zn^2+^ in the glass network because of the larger electronegativity of Mo^6+^. Accordingly, the α increased for the TZB–Mo glass system for different MoO_3_ contents.

The trends of individual series can also be explained based on the conversion of TeO_4_ → TeO_3+δ_ → TeO_3_. From the Raman spectra, as shown in Figure 2, for TZB–Mo series glasses, there is a rapid transformation of TeO_4_ tbp units into TeO_3_ for the substitution of MoO_3_ modifier content. This change is associated with the intermediate TeO_3+δ_ state, which possesses an elongated axial bond and further, because the elongation of this bond is significant with respect to the shortening of the equatorial bond lengths, the increase in α can be understood in terms of a decrease in bond energy associated with the creation of TeO_3+δ_ subunits. Conversely, it was worthy to note that the Nb_2_O_5_-modified TZB glasses showed lower α and higher T_dg_ and T_ds_ than that of TZB- and MoO_3_-based TZB glass systems, indicating a candidate material for the glass molding process.

### 3.5. Mechanical Studies

**Figure 7:**
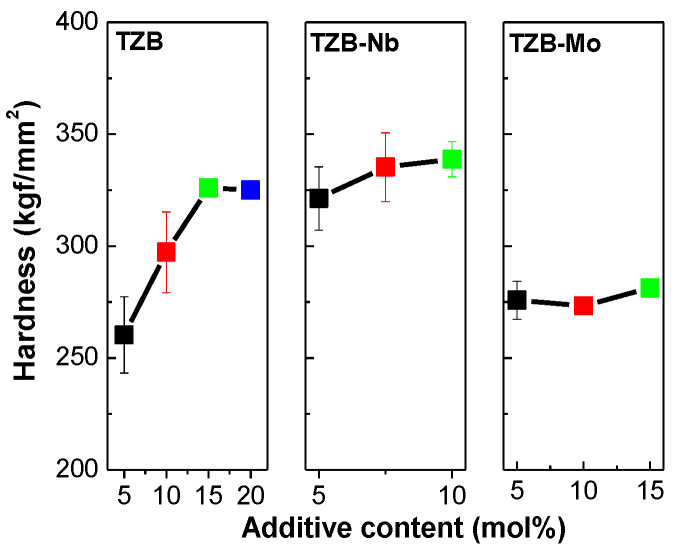
Knoop hardness (H_K_) of the TeO_2_–BaO–ZnO glass system varied with different additive concentrations.

Micro-indentation is a common test to determine the physical properties of glasses for specific application. As per previous literature, the tellurite-based glasses have been reported to be very weak, and therefore, a Knoop indenter can be used to measure the hardness of glasses [59,60]. The Knoop hardness (H_K_) can be obtained using the following equation [61], given by:

(5) $$H_{K}=\frac{P}{L^{2}C_{p}}$$

where P is the applied load in Newton (N), L is the length of indentation diagonal (μm) and C_p_ is the correction factor (0.07028). Figure 7 depicts the variation of H_K_ with respect to glass composition at different concentrations of modifiers. As can be seen from the figure, the H_K_ values were found to increase from 260 to 325 kgf/mm^2^ with the addition of BaO content that was increased from 5 to 20 mol%, respectively. The H_K_ values were found to be 321, 335 and 339 kgf/mm^2^ for different Nb_2_O_5_ concentrations of 5, 7.5 and 10 mol%, respectively. The H_K_ values were determined to be 276, 273 and 281 kgf/mm^2^ for different MoO_3_ concentrations of 5, 7.5 and 10 mol%, respectively. From the results, it was noticed that the hardness increased with respect to modifier concentration. It was worthy to note that the Nb_2_O_5_-modified tellurite glasses had higher H_K_ values that that of other studied TZB and TZB–Mo glass systems. The results direct that the TZB–Nb glasses have much larger mechanical strength than that of TZB and TZB–Mo glasses.

## 4. Conclusions

In the present study, all three series TZB glasses modified with different additives of BaO, Nb_2_O_5_ and MoO_3_ at different concentrations were prepared by the melt-quenching technique and their structural, physical and optical properties were studied systematically for mid-infrared transmitted optical glass material. The Raman studies of the present glasses resemble the basic structural behavior of tellurite glasses. The structural modification was noted in the TeO_2_ network with the additions of Nb_2_O_5_ and MoO_3_ modifiers. The bands at ~860 and 900 cm^−1^ were assigned to the ν_s_(Nb–O) stretching vibrations in NbO_6_ octahedra and Mo=O vibrations in MoO_6_ octahedra, respectively. The studied glasses were transparent in the mid-IR region covering from 3 to 6 μm, and the maximum transmittance of about 70% was noticed for the Nb-doped glass system. High refractive index of the order of 1.99 at 3 μm was obtained for the Nb_2_O_5_-based glass. The glass transition temperature was determined to be ~360, 409 and 352 °C for TZB, TZB–Nb and TZB–Mo glass systems, respectively. From the thermal and mechanical studies, it was concluded that the Nb_2_O_5_-based TZB glass system exhibited a high stability of 192 °C, low CTE of 11.35 × 10^−6^/K at 100 °C and high Knoop hardness of 339 kgf/mm^2^. The results of the studied glasses were compared to the commercial SUMITA optical glasses. It was noticed that the Nb_2_O_5_-based TZB glasses are promising for the application of mid-wave infrared optical glass lenses.

## Figures and Tables

**Table 1:** Glass compositions and their labels of TeO_2_–ZnO–BaO glasses.

S. No.	Glass Label	Molar Composition (mol%)
1	TZB1	65TeO_2_–30ZnO–5BaO
2	TZB2	60TeO_2_–30ZnO–10BaO
3	TZB3	55TeO_2_–30ZnO–15BaO
4	TZB4	50TeO_2_30ZnO–20BaO
5	TZB–5Nb	60TeO_2_–25ZnO–10BaO–5Nb_2_O_5_
6	TZB–7.5Nb	60TeO_2_–22.5ZnO–10BaO–7.5Nb_2_O_5_
7	TZB–10Nb	60TeO_2_–20ZnO–10BaO–10Nb_2_O_5_
8	TZB–5Mo	60TeO_2_–25ZnO–10BaO–5MoO_3_
9	TZB–10Mo	60TeO_2_–20ZnO–10BaO–10MoO_3_
10	TZB–15Mo	60TeO_2_–15ZnO–10BaO–15MoO_3_

**Table 2:** Raman bands’ frequency and their assignment in the TZB series glass system for different modifier contents.

Optical Phonon Frequency, ν (cm^−1^)										Assigned Vibrational Modes
TZB				TZB–Nb			TZB–Mo			
1	2	3	4	5 Nb	7.5 Nb	10 Nb	5 Mo	10 Mo	15 Mo	
309	300	301	304	308	310	310	304	326	338	ν(Te–O) stretching and bending vibrations in TeO_3+δ_ and TeO_3_ networks
428	438	437	437	439	440	440	433	436	436	ν_s_(Te–O–Te) stretching and bending vibrations
671	675	683	683	681	674	676	678	678	679	ν_as_(Te–O) stretching vibrations in TeO_4_ networks
754	759	769	769	771	762	758	754	750	745	ν(Te–O) stretching vibrations in TeO_3_ and TeO_3+δ_ networks
-	-	-	-	865	860	857	900	905	909	ν_s_(Nb–O) stretching vibrations of bonds in NbO_6_ octahedra

**Table 3:** The glass transition temperature (T_g_), onset crystallization temperature (T_x_), glass stability parameter (∆T), dilatometric glass transition temperature (T_dg_), dilatometric softening temperature (T_ds_), coefficient of thermal expansion (α) and Knoop hardness (H_K_) of TeO_2_ glasses modified with different contents of BaO, Nb_2_O_5_ and MoO_3_ at various concentrations.

Glass Sample	T_g_ ±2 (°C)	T_x_ ±2 (°C)	∆T (°C)	T_dg_ ±2 (°C)	T_ds_ ±2 (°C)	α ±0.1 (×10^−6^/K)	H_K_ (kgf/mm^2^)	Reference
TZB1	336	442	106	356	377	14.15	260	This work
TZB2	344	483	139	364	384	14.49	297	This work
TZB3	353	507	154	374	394	14.74	326	This work
TZB4	360	509	149	380	398	15.08	325	This work
TZB–5Nb	388	580	192	399	431	12.75	321	This work
TZB–7.5Nb	400	576	176	384	399	12.40	335	This work
TZB–10Nb	409	598	189	422	454	11.35	339	This work
TZB–5Mo	350	487	137	357	370	14.63	276	This work
TZB–10Mo	351	471	120	360	375	14.90	273	This work
TZB–15Mo	352	408	56	360	377	14.98	281	This work
